# Novel static magnetic field effects on green chemistry biosynthesis of silver nanoparticles in *Saccharomyces cerevisiae*

**DOI:** 10.1038/s41598-021-99487-3

**Published:** 2021-10-11

**Authors:** Ameni Kthiri, Selma Hamimed, Abdelhak Othmani, Ahmed Landoulsi, Siobhan O’Sullivan, David Sheehan

**Affiliations:** 1 Laboratory of Biochemistry and Molecular Biology, Carthage University, Faculty of Sciences of Bizerte, Zarzouna, Bizerte, Tunisia; 2 Laboratory of Material Physics: Structures and Properties, LR01 ES15, University of Carthage, Faculty of Sciences, Zarzouna, 7021 Bizerte, Tunisia; 3 Department of Molecular Biology and Genetics, College of Medicine and Health Sciences, Khalifa University of Science and Technology, Abu Dhabi, United Arab Emirates; 4 School of Biochemistry and Cell Biology, University College Cork, Cork, Ireland; 5 Department of Chemistry, College of Arts and Sciences, Khalifa University of Science and Technology, PO Box 127788, Abu Dhabi, United Arab Emirates

**Keywords:** Biophysics, Biotechnology, Chemistry, Materials science, Nanoscience and technology

## Abstract

The bacteriocidal properties of silver nanoparticles (AgNPs) depend on their average diameter (toxicity increases with decreasing diameter). In the present work, we describe novel green chemistry biosynthesis of AgNPs from AgNO_3_ added to cell-free culture medium of baker’s yeast, *Saccharomyces cerevisiae*, yielding nanoparticles in the range 11–25 nm. However, when yeast was grown in a moderate static magnetic field, AgNPs obtained from the resulting cell-free culture medium, were significantly smaller (2–12 nm) than those obtained without magnetic field. These latter nanoparticles were highly crystalline, stable and near-uniform shape. Furthermore, the antibacterial activity of AgNPs obtained from static magnetic fields were greater than those from control cultures. Static magnetic fields show a promising ability to generate biocidal nanoparticles via this novel green chemistry approach.

## Introduction

Green chemistry proposes use of environmentally sustainable routes to the design, manufacture and application of useful chemical products. The aim is to reduce production or accumulation of potentially harmful reagents with associated health and environmental risks^1^. Silver nanoparticles (AgNPs) are potent antimicrobials and, commercially, are the most quantitatively important category of metallic nanoparticles—in turn the most important category of engineered nanomaterial^2^. Their conventional synthetic routes are expensive and require toxic solvents and by-products. It can also be difficult to control nanoparticle size-distribution. A popular green chemistry approach to metal nanoparticle synthesis is to exploit biological systems which can reduce metals leading to nanostructures. This has been achieved with aqueous extracts of bacteria, fungi, plants, and waste products possessing efficient reducing and stabilization systems^3–5^. Intact plant and fungal cells have also shown an ability to reduce metals either intracellularly or extracellularly^6^. A variety of plant and microbial systems have been used to prepare AgNPs including seaweed, curry leaf, *Aspergillus, Fusarium* and lactobacilli^7^. These approaches are scalable, inexpensive and environmentally benign^8^. The precise mechanism of reduction is poorly-understood but some cells contain or secrete reductase enzymes and/or reducing agents, which may provide biochemical routes to metal reduction. Metal and metal oxide nanoparticles are useful in a wide variety of commercial applications in consumer products. These exploit their unusual electrical, optical, stability and catalytic properties^9^. Because of their potent antimicrobial activity, AgNPs are incorporated in antimicrobial soaps, wound-dressings, creams and biomedical devices such as catheters and valves^10,11^ which are especially susceptible to growth of bacterial biofilms.

Static magnetic fields (SMF) are time-independent fields of constant strength, which arise in the environment from a variety of sources including the Earth’s own magnetic field, direct current transmission lines and domestic electrical devices (e.g. microwaves and electronics). Lifestyle-related exposure sources include magnetic resonance imaging, occupational exposure (e.g. welding and audiovisual units) and personal electronic devices such as mobile phones^12^. It is difficult to shield against SMFs so they can readily penetrate biological material and interact with charged species such as ions and proteins^13,14^. The interactions between biology and magnetic fields remain somewhat mysterious. However, studies have shown profound effects of SMF on mice including oxidative stress and weight loss^15^. Investigation of the effect of SMF on cultured mammalian and microbial cells also indicated an increase in oxidative stress^16^. A recent study demonstrated that baker’s yeast, *Saccharomyces cerevisiae*, experiences oxidative stress and profound reduction in growth rate in the presence of weak SMF^17^. Here, we explore a novel green chemistry approach to biosynthesis of AgNPs using cell-free supernatants of *S. cerevisiae* cultures grown in the presence and absence of SMF. When silver nitrate was added to cell-free culture supernatants, biosynthesis of AgNPs resulted. Interestingly, supernatants of cultures grown in the presence of SMF produced notably smaller AgNPs which were more bacteriocidal. To our knowledge, this is the first report on the effects of SMF on the biosynthesis of metal nanoparticles.

## Results and discussion

**Figure 1:**
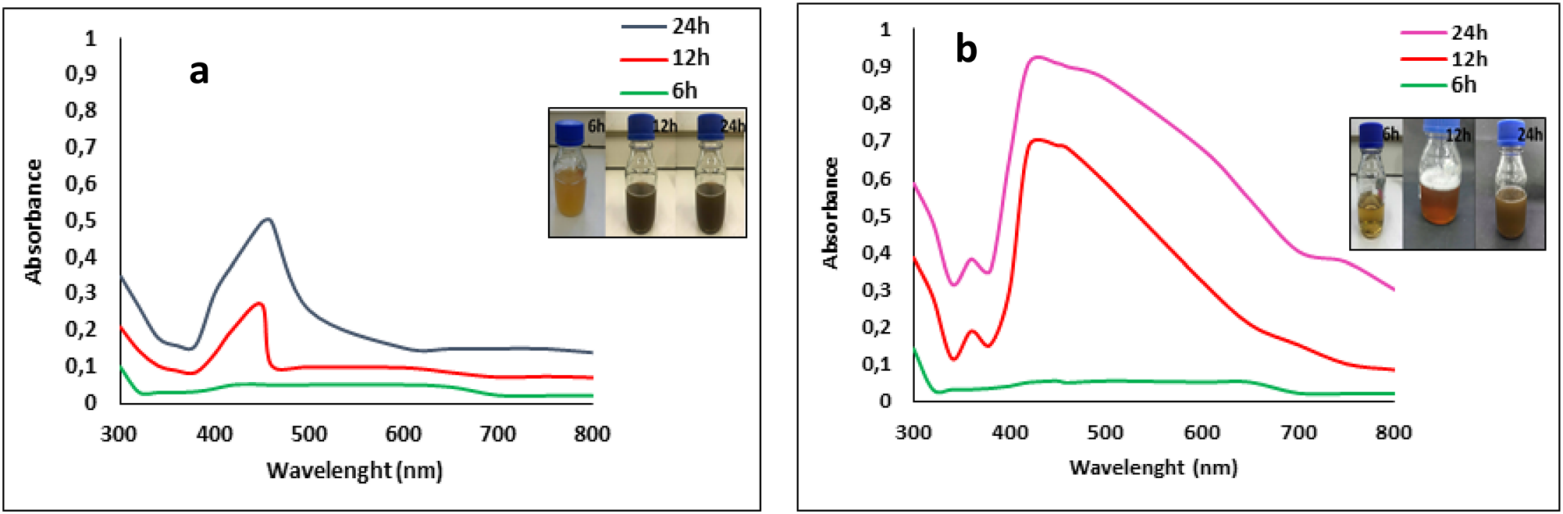
UV–vis spectra of biosynthesized AgNPs recorded at time intervals 6, 12 and 24 h. **(a)** AgNPs obtained with supernatant from non-exposed cultures (AgNPs_CONTROL_), **(b)** AgNPs obtained with supernatant from SMF-exposed cultures (AgNPs_SMF)._ Inset to **(a,b)**; the intensity of yellow to brown color change increases with time (6 h, 12 h and 24 h).

In the present study, *S. cerevisiae* culture was used for the synthesis of AgNPs. The color of *S. cerevisiae* control supernatant changed from light yellow to brown with the addition of 1 mM aqueous solution of silver nitrate after 24 h of incubation at 30 °C (Fig. 1a inset). When the *S. cerevisiae* culture that had been exposed to SMF was treated with 1 mM AgNO_3_, the color of the reaction medium gradually intensified with increased incubation period time as shown in Fig. 1b, it changed from yellow to reddish brown (12 h at 30 °C) and then dark brown (24 h at 30 °C). These color changes were consistent with synthesis of nanoparticles. Previously, it has been reported that the reduction of Ag^+^ into AgNPs can be seen very clearly when the color of the solution changes from yellow to brown due to the excitation of surface plasmon resonance (SPR)^18,19^.

To follow the process of Ag reduction, mixtures were scanned in a UV–Vis spectrophotometer at 6 h, 12 h, and 24 h (Fig. 1). Absorbance maxima at 420 and 450 nm for AgNPs_SMF_ and AgNPs_control_, respectively, were observed and intensity increased with incubation time. This intensity increase was consistent with reduction of silver ions and an increase in the concentration of AgNPs^20^. It seems that nucleation was initiated more rapidly and the formation of AgNPs occurred more quickly by using a yeast filtrate from cultures exposed to SMF as opposed to the filtrates from control cultures (no SMF). The absorption band at around 265 nm, has previously been attributed to aromatic residues in proteins^21^. It is believed that metabolic products of the yeast are mainly responsible for the reduction of Ag^+^ ions.

**Figure 2:**
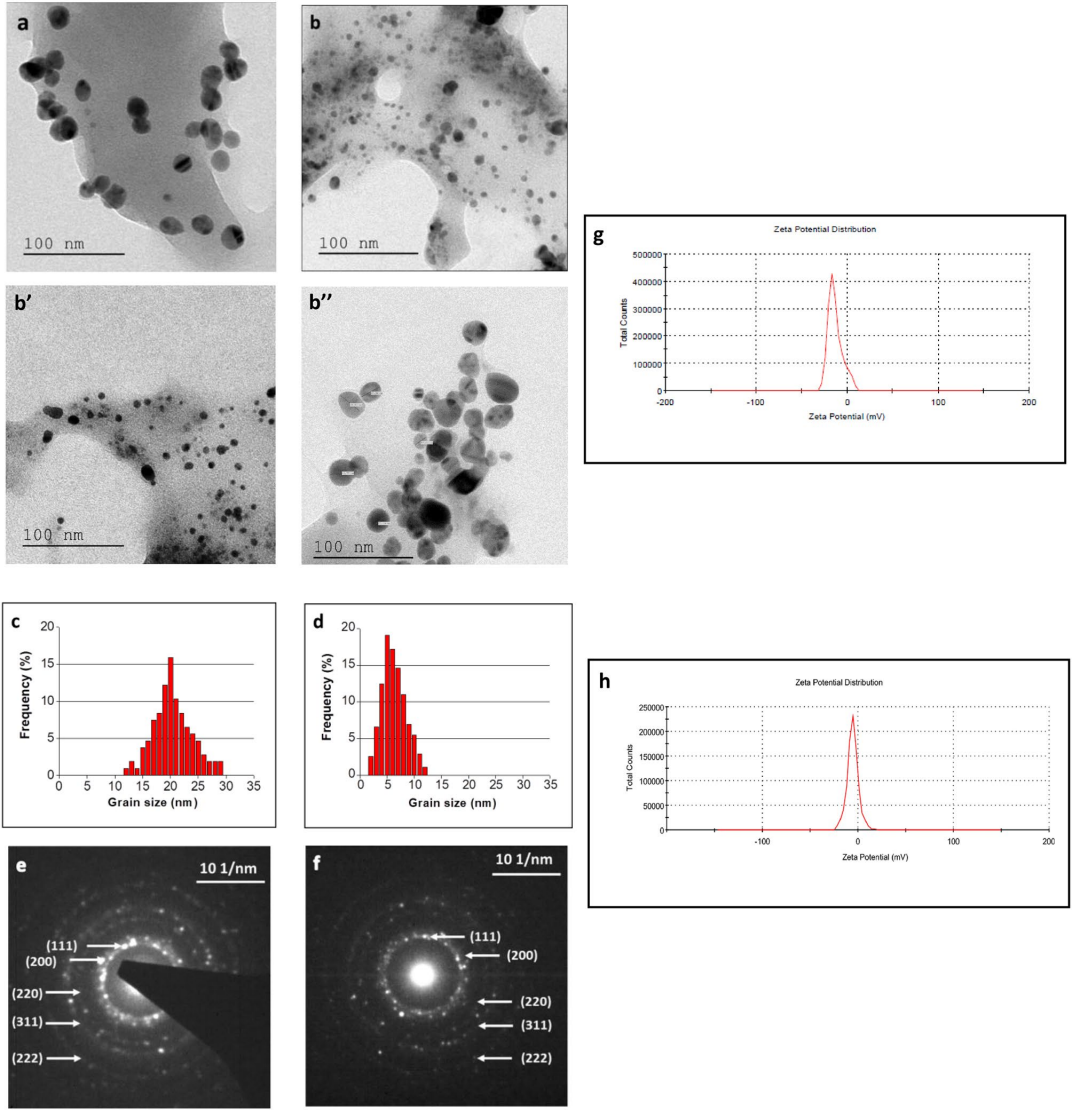
Biosynthesis of AgNPs. Scanning electron micrograph of AgNPs formed in **(a)** control culture supernatant and in supernatant of culture grown in presence of **(b,b’,b’’)** 250mT SMF. Histograms of AgNP average diameter size formed in **(c)** control culture supernatant and in supernatant of culture grown in presence of **(d)** 250mT SMF. Selected-area electron diffraction (SAED) patterns for AgNPS from **(e)** control and **(f)** SMF culture supernatants. Zeta potential of AgNPs formed in **(g)** control culture supernatant and in supernatant of culture grown in presence of **(h)** 250mT SMF.

Media from yeast cultures grown in the presence and absence of SMF produced small AgNPs on the addition of AgNO_3_ while blank medium (no yeast cells) did not show any nanoparticle synthesis. When analyzed by TEM, AgNPs were in the diameter ranges 11–25 nm and 2–12 nm for media from cultures grown in the absence and presence of SMF, respectively (Fig. 2a, b, b’, b’’). The nanoparticles were mostly spherical in both cases but those from SMF-exposed cultures seemed to be more uniform (Fig. 2b’,b’’). All nanoparticles were well-separated and no agglomeration was noticed. Histograms of average diameters showed quite narrow size distributions of the synthesized AgNPs without and under magnetic field (Fig. 2c,d). The nanoparticles were highly crystalline as shown by selected-area electron diffraction (SAED) patterns (Fig. 2e,f).

Niknejad et al*.*^22^ reported that biosynthesis of AgNPs by *S. cerevisiae* resulted in mainly spherical and polydispersed nanoparticles with diameters in the range 5–20 nm. Korbekandi et al*.*^23^ made AgNPs from dried culture of *S. cerevisiae* with a spherical shape and diameter range of 2–20 nm. In our study, the diameters of the obtained AgNPs were smaller than in these reported and also than those synthesized from *Candida utilis* (20–80 nm)^24^. To the best of our knowledge, there are no previous reports on using cell-free cultures or SMF to modify biosynthesis of nanoparticles in yeast cultures.

Moreover, the zeta potential is an important indicator of the surface charge of synthesized nanoparticles in colloidal dispersion. In Fig. 2g,h, the surface charge of AgNPs_CONTROL_ and AgNPs_SMF_ were around – 13.2 mV and – 10 mV, respectively. These results indicate the stability of the silver nanoparticles, which are highly polydispersed due to their negative charge^25^.

Concentric rings with intermittent bright dots were evident in SAED, indicating a high degree of crystallinity (Fig. 2e,f). The lattice constant calculated from this pattern was a = 4.087 Å, which agrees well with the reported value of 4.086 Å^18^. A fringe spacing of 0.23 nm was measured between lattice planes and is in accordance with the (1 1 1) lattice spacing of face-centered cubic (fcc) silver (d1 1 1 = 0.2359 nm). Average particle diameters of AgNPs were calculated from X-ray diffraction using the Debye–Scherrer equation^26^ as 18 nm (control) and 6 nm (SMF), respectively (Fig. 2a,b). These values agree well with those from TEM (Fig. 2c,d).

**Figure 3:**
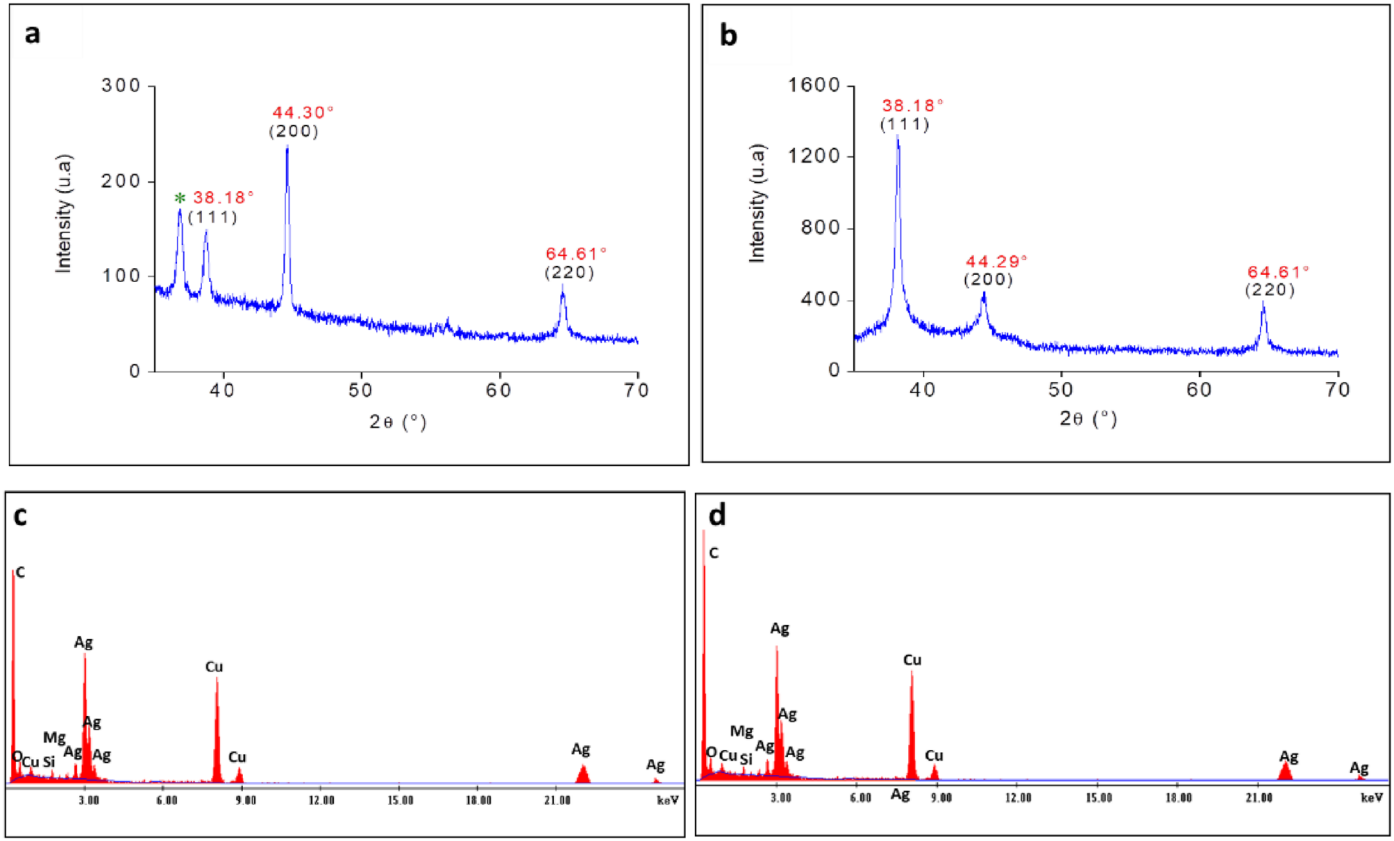
Characterization of AgNPs. X-ray diffraction of AgNPs formed in **(a)** control culture supernatant and in supernatant of culture grown in presence of **(b)** 250 mT SMF. Elemental composition of AgNPs formed in **(c)** control culture supernatant and in supernatant of culture grown in presence of **(d)** 250 mT SMF.

Crystallinity of the biosynthesized nanoparticles was evaluated using X-ray diffraction (XRD). Patterns presented in Fig. 3 show several Bragg reflections with various 2θ values depending on the AgNPs batches. Nevertheless, both AgNPs formulations exhibited three diffraction peaks (marked with asterisks in Fig. 3a,b), at 2θ: 38.28°, 44.33° and 64.33°, which correspond to the Bragg reflections of (111), (200) and (220) planes of face-centered cubic lattice structure of metallic silver (JCPDS-ICDD files No 04-0783). This confirms the successful synthesis of AgNPs and establishes their crystalline nature^27,28^. Similar diffraction patterns were observed with AgNPs biosynthesized using other yeast and plant extracts^29–32^. These data confirmed the purity of the synthesized nanoparticles.

Elemental composition on the surface of the biosynthesized nanoparticles was determined using EDX spectroscopy. In general, the optical absorption peak of silver appears at around 3 keV due to SPR^33,34^. As shown in Fig. 3c,d, EDX analysis revealed the presence of silver in the nanoparticles formed from SMF-treated cultures and those of controls. Silver peaks were the most prominent for all the samples, but no nitrogen peaks were evident in the EDX patterns of the AgNPs formulations. This indicates that no ions from AgNO_3_ were present in AgNPs, consistent with complete reduction of silver ions as indicated by UV–Vis spectroscopy and visual observation. In addition to silver peaks, EDX revealed the presence of carbon and copper atoms on the surface of the biosynthesized AgNPs that may be due to the TEM grid composition. We also noticed that these silver nanoparticles were accompanied by different trace impurities magnesium (Mg) and silicon (Si). It is known that organisms such as yeasts, fungi, and plant metabolites have previously been reported to act not only as reducing agents but also as capping agents for AgNPs^35,36^. Herein, it could be hypothesized that the extracellular synthesis of AgNPs occurs due to the adsorption of silver ions on the surface of metabolic products (enzymes, polysaccharides, compound derived magnesium or silicon, etc.) present in the culture supernatant of *S. cerevisiae*, which reduce the Ag^+^ to AgNPs. While, the effects of SMF on the synthesis of nanoparticles have not yet been elucidated, it is believed that SMF creates wave cavitation through the liquid that may enhance the decomposition of biomolecules and liberate free radicals, which then act as reducing agents.

**Figure 4:**
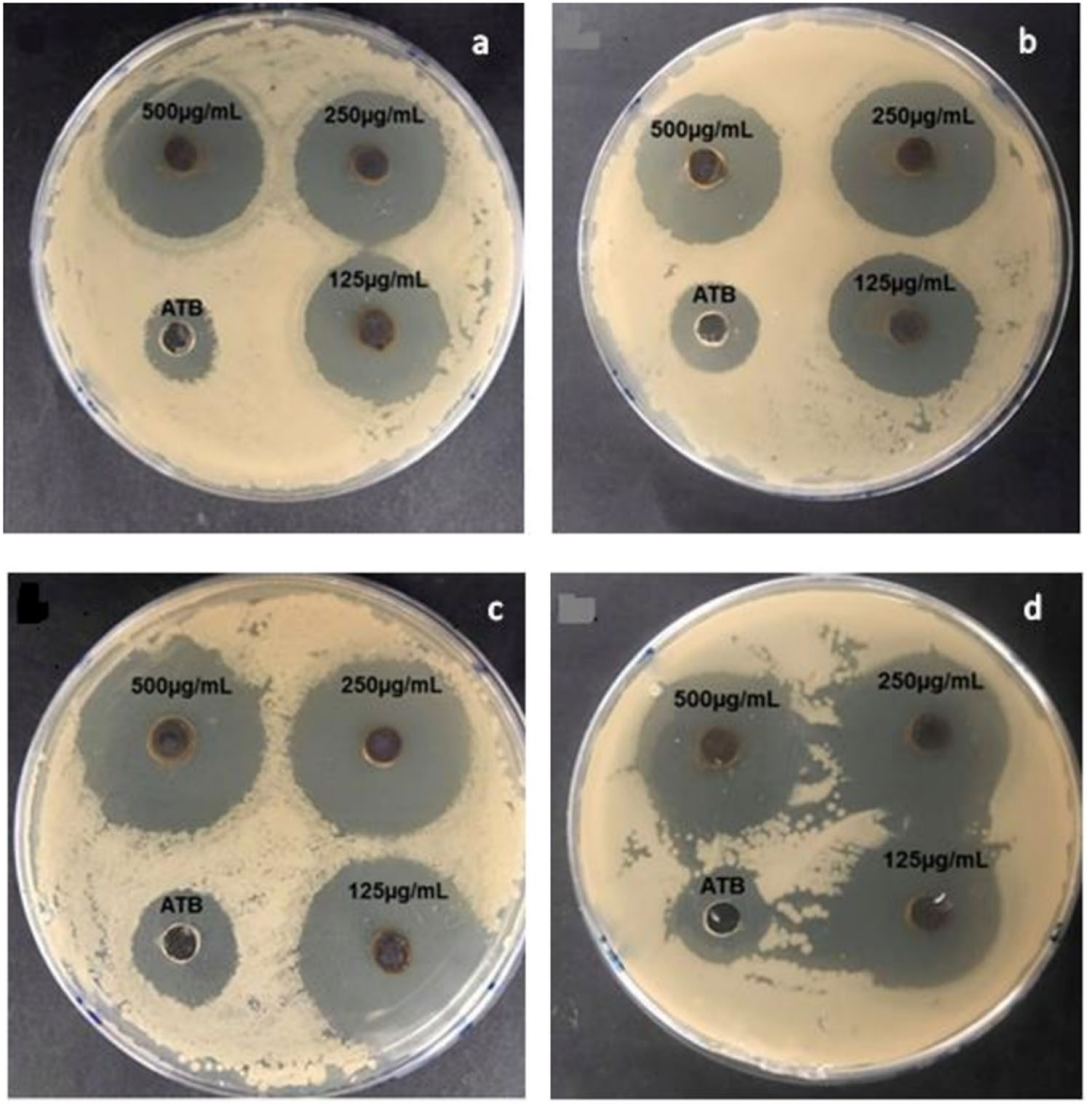
Antibacterial activity of biosynthesized AgNPs at different concentrations (125, 250, and 500 µg/mL) evaluated by agar well diffusion. AgNPS_Control_ against **(a)** *E. coli* (**b)** *S. aureus*. AgNPs_SMF_ **(c)** *E. coli* **(d)** *S. aureus*.

Antibiotic resistance of human pathogens is an important public health concern. Nanoparticles may provide a viable alternative to conventional antibiotics^37^. The biosynthesized AgNPs, in our study, showed effective inhibition of bacterial growth (Table 1 and Fig. 4). Their respective minimum inhibitory concentration (MIC) and minimum bactericidal concentration (MBC) were 25 µg/mL and 70 µg/mL for AgNPs_SMF_; 40 µg/mL and 90 µg/mL for AgNPs_CONTROL_ for *E. coli*. While, *S. aureus* showed the respective value of MIC and MBC: 15 µg/mL and 50 µg/mL for AgNPs_SMF_; 20 µg/mL and 70 µg/mL for AgNPs_CONTROL_ These results suggest that the AgNPs prepared from cultures grown in SMF displayed good to excellent inhibitory effects as antibacterial agents, due to their smaller size. Generally, the difference in the peptidoglycan layer of the bacterial cell between Gram ( +) and Gram (–) bacteria plays a crucial role in the inhibitory activity^38–40^. In addition, the negative surface charge and low size of AgNPs are found to be effective factors enhancing antibacterial activity^41^.

**Table 1:** Antibacterial activity of biosynthesized silver nanoparticles (AgNPs) at different concentrations (125, 250, and 500 µg/mL) against *E. coli* and *S. aureus*. Data represent mean ± SD (n = 3).

Mean width of inhibition zone (mm)		
	*E. coli*	*S. aureus*
Ciprofloxacin	17.16 ± 0.68	16.16 ± 0.68
**Concentration(125 µg/ml)**		
AgNPs_CONTROL_ AgNPs_CMS_	24.66 ± 1.36 28 ± 1.78	20.27 ± 0.62 25.6 ± 0.58
**Concentration(250 µg/ml)**		
AgNPs_CONTROL_ AgNPs_CMS_	25.1 ± 0.93 31 ± 0.89	23 ± 0.89 25.66 ± 0.58
**Concentration(500 µg/ml)**		
AgNPs_CONTROL_ AgNPs_CMS_	28.66 ± 1,03 33.66 ± 1.36	24.66 ± 1.36 29 ± 0.89

Smaller AgNPs show the highest antibacterial effect and are more cytotoxic when compared to larger nanoparticle diameters. This may be due to easier absorption and/or a larger functional surface area^42,43^. It has been reported that the concentration and small size of AgNPs play important roles in antimicrobial activity by easier diffusion, penetration into bacterial cell membranes and growth inhibition^44,45^. In addition, the AgNPs induce the generation of reactive oxygen species inside bacterial cells that damage the membrane leading to cell death^46^.

**Figure 5:**
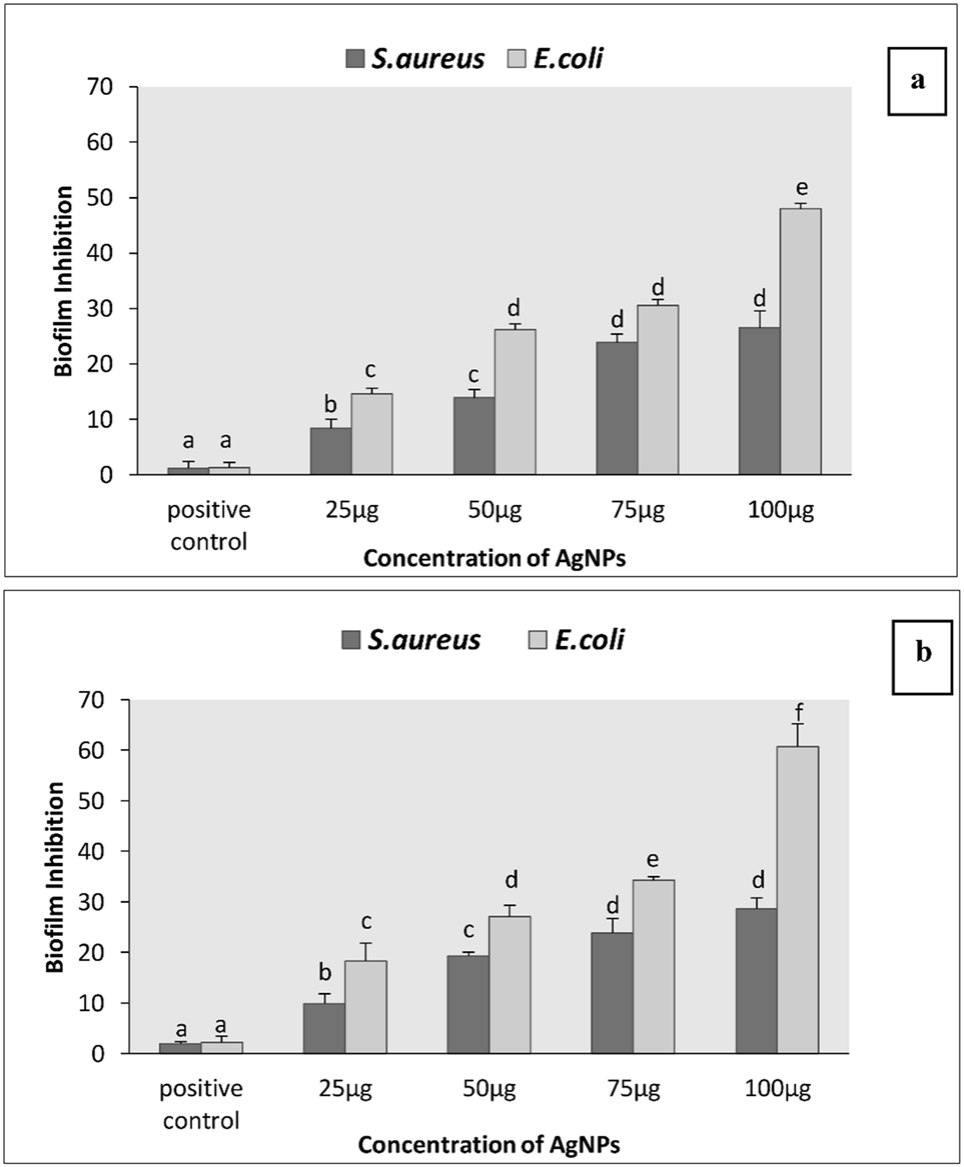
Determination of the rate of antibiofilm inhibition by biosynthesized AgNPs **(a)** AgNPs_CONTROL,_ **(b)** AgNPs_SMF_. The results are expressed as means + /– SD of triplicate measurements. Significant differences are indicated by different letters (b, c, d, e, f) vs. positive control (p < 0.05).

Antimicrobial agents that are effective against free-living bacterial cells, are sometimes ineffective against the same species growing in a biofilm state. AgNPs have previously been shown to be potentially important candidates against biofilms^47,48^. AgNPs prepared in this study showed good ability to inhibit biofilm formation. Tested microorganisms were grown in microliter plate wells with/without AgNPs to form a biofilm for 24 h. The treatment of cell-free filtrate (positive control) showed no significant decrease in biofilm formation (Fig. 5). An effective antibiofilm activity of both AgNPs against Gram (–) and Gram ( +) bacteria was observed, which may be due to the easy diffusion of AgNPs inside cells damaging the biofilm metabolic formation^49^.

It is evident that the Ag-NPs_SMF_ exhibited greater antibiofilm activity than the Ag-NPs_control_. This difference in inhibitory activity of both AgNPs can be explained by several factors, including efficacy in antimicrobial activity, physical and chemical properties such as affinity between the AgNPs and biofilms^50^. Goswami et *al.*^51^ previously investigated AgNPs-mediated biofilm eradication and they found an inhibition of 89% for *S. aureus* and 75% for *E. coli* at 15 mg/mL. Similarly, Barapatre et *al.*^52^ reported a significant decrease of biofilm formation at (64 µg/mL) of 70% and 30% for Gram (–) bacteria and Gram ( +) bacteria, respectively. In addition, Rolim et *al.*^53^ demonstrated a potential antibiofilm activity of biologically synthesized AgNPs against *S. aureus* and *E. coli,* which agree with our findings. This supports application of AgNPs from yeast culture supernatants as biofilm-disrupting agents.

The present study demonstrates the efficiency of yeast (*S. cerevisiae*) cultures for green chemistry biosynthesis of bacteriocidal nanoparticles. Employing SMF promoted rapid formation of AgNPs with smaller particle diameter which were more highly crystalline than those from control cultures. Biosynthesized AgNPs formed under both conditions displayed pronounced antimicrobial and antibiofilm activities with two model pathogenic microorganisms. We hypothesize that yeast cells stressed by SMF secrete reductases and/or reducing agents in response to oxidative stress^17^. These likely promote more efficient reduction of silver forming AgNPs. SMF provides a promising and completely novel route to green chemistry biosynthesis of commercially important nanoparticles.

## Materials and methods

### Growth and treatment of yeast cells

*S. cerevisiae* (strain BY4741. Genotype: *MATa his3*Δ*1 leu2*Δ*0 met15*Δ*0 ura3*Δ*0 o*btained from the Euroscarf collection; http://www.euroscarf.de) were cultured in YPD broth (1% bacto-yeast extract, 2% bacto-peptone, 2% glucose). The cultures were exposed to a parallel and globally homogeneous magnetic induction of 250 mT and SMF was generated by a pair of cylindrical coils (diameter 20 cm, length 13 cm; Beaudouin, Paris, France). The terrestrial magnetic field at the experimental location was 0.075 mT. Control cultures were prepared identically but with no SMF. Cell-free supernatants were obtained from each culture by centrifugation (10,000*g*) and 1 mM AgNO_3_ was added to this supernatant. The supernatants were maintained in the dark at room temperature for 24 h. AgNPs were collected by sequential centrifugation.

### Characterization of nanoparticles

#### UV–visible absorbance spectral analysis

The biosynthesized AgNPs were preliminarily detected by visual observation of the color change of cell filtrate after treatment with silver nitrate (1 mM). Characterization of the synthesized Ag-NPs was further carried out using scanning absorbance spectra (200–800 nm) of the mixture using a UV–Visible spectrophotometer (Biowave II (England)) with a resolution of 1 nm.

#### Transmission electron microscopy (TEM)

The transmission electron microscopy (TEM, JEOL JEM-1010) operated at 200 kV accelerating voltage was performed on the biosynthesized AgNPs. 200 kV accelerating voltage was used to investigate the process of formation of AgNPs, and to study their sizes and shapes. Samples for TEM were prepared by drop-coating the AgNPs suspensions onto carbon-coated copper grids. Micrographs were obtained using an EM 10C ZEISS^®^ (Germany) TEz.

#### Energy dispersive X-ray (EDX)

EDX analysis was also performed by energy-dispersive spectroscopy (EDS) using INCA Energy TEM 200 with analysis software (JEOL) for identifying the elemental composition of the biosynthesised AgNPs.

#### X-ray diffraction analysis (XRD)

Films of nanoparticle sizes and their crystalline structure were examined using an X-ray diffractometer (Bruker D8 ADVANCE). The operating voltage of 40 kV and current of 30 mA were used with Cu kα radiation of 0.1541 nm wavelength, in the 2θ range 10°–80° angle.

#### Zeta potential

The zeta potential measurement was used to determine the surface potential of the synthesized AgNPs in water was analyzed by Malvern Nano-Zetasizer ZS (Malvern, UK).

### Antimicrobial properties of nanoparticles

Antibacterial activity of AgNPs was evaluated against*, E. coli* and *S. aureus* using the agar-well diffusion and broth micro dilutions method^1^. Approximately 10^7^ colony forming units (CFU/mL) of each strain were inoculated in Muller-Hinton agar (MHA) plates and then 50 μl of AgNPs solution with different concentrations (125, 250, and 500 µg/mL) was added to wells which were bored using a core borer of 6 mm diameter. 1 mM silver nitrate solution and ciprofloxacin antibiotic were used as controls. The plates were incubated at 37 °C for 24 h. After incubation, the maximum zone of inhibition was observed and measured for analysis against each type of test. Moreover, stock solutions of tested AgNPs were prepared by serial twofold dilution to obtain a serial concentration ranging from 10 to 100 µg/mL. An aliquot of working suspension (100µL) was added to a sterile 96-well plate containing (100 µL) of tested stock solutions. Plates were incubated for 24 h at 37 °C. According to the Lehtinen et al.^54^ method, the minimum inhibitory concentration (MIC) was defined as the lowest concentration of the tested compounds that completely inhibited microbial growth and the minimum bactericidal concentration (MBC) were recorded as the lowest concentration giving no visible microorganisms. The standard error was calculated using three experimental replicates.

Antibiofilm activity of AgNPs with *S. aureus* and *E. coli* was tested in order to determine ability to inhibit biofilm formation using 96-well microtiter plate method^55^. Individual wells of the sterile microliter plate were filled with 180 μL of MH broth, inoculated with 10 μL of overnight grown culture of each strain and 10 μL of AgNPs solution at various concentrations (25, 50, 75, and 100 µg). The microliter plates were incubated for 24 h at 37 °C. Only sterile Muller Hinton Broth was used as the blank. After incubation, content of each well was gently removed and washed with 0.2 mL of phosphate buffered saline (PBS, pH 7.2) three times, to remove free-floating ‘planktonic’ cells. The remaining biofilms in the wells were mixed with 2% sodium acetate (SigmaAldrich S2889) and stained with 0.1% crystal violet. Then, the excess dye was washed with deionized water and the wells were kept for drying. After drying, 200 μL of 95% (v/v) ethanol was added to the wells. After incubation, content of each well was gently removed and washed with 0.2 mL of PBS (pH 7.2) three times, to remove free-floating ‘planktonic’ bacteria. Biofilms formed by adherent ‘sessile’ organisms in plate walls were fixed with sodium acetate (2%, w/v) and stained with crystal violet dye (0.1%, w/v). Excess stain was rinsed off by thorough washing with sterilized Millipore water and plates were kept for drying. After drying, 200 μL of 95% (v/v) ethanol was added to the wells.

The absorbance was measured at 620 nm on an ELISA reader (Multiskan® EX, Thermo Scientific, Finland) and values obtained were considered as an index of bacteria adhering to the surface of well wall for developing biofilms. The percentage of biofilm inhibition was calculated using the following equation:

$$ \% \, Inhibition \, of \, biofilm \, = \frac{{\left( {OD \, of \, control \, {-} \, OD \, of \, treatment} \right)}}{OD \, of \, treatment} \times 100. $$

### Statistical analysis

All antibacterial and antibiofilm activities were carried out in three independent determinations and all data are presented as mean ± standard deviation. The significance of difference between mean values and comparison among groups was performed by using the one-way ANOVA test followed by Tuckey’s test with SPSS software version 25. Values of p < 0.05 was considered statistically significant.

## Conclusion

Media from cultures of *S. cerevisiae* were prepared by removing yeast cells and proved capable of synthesizing AgNPs from AgNO_3_ without additional chemical agents. This use of yeast culture medium provides a novel, scalable and green chemistry route to produce biocidal AgNPs with narrow size-ranges, crystalline shape, and spherical form. However, medium from cells grown in the presence of moderate SMF biosynthesized significantly smaller AgNPs with more potent antibacterial and antibiofilm activities. AgNPs produced in this way were physically characterized by a variety of methods and found to be of excellent quality. Our approach provides a novel green chemistry route to synthesis of metallic nanoparticles with important biomedical and commercial applications. We speculate that chemical reducing agents are secreted into culture medium more abundantly from cells treated with SMF leading to efficient reduction of silver to AgNPs.
